# Outcomes of Dynamic Condylar Screw Fixation for Unstable Peritrochanteric Fractures

**DOI:** 10.7759/cureus.22866

**Published:** 2022-03-05

**Authors:** Gauhar N Khan, Hassan R Khosa, Muhammad Usman, Jahanzeb Mazari, Irfan Qadir

**Affiliations:** 1 Trauma and Orthopaedics, Recep Tayyip Erdogan Hospital-The Indus Hospital, Muzaffargarh, PAK

**Keywords:** unstable peritrochanteric fractures, subtrochanteric fractures, pakistan, dynamic condylar screw, proximal femur fractures

## Abstract

Background

The optimal treatment modality and choice of implant for unstable peritrochanteric fractures is debatable, with multiple options ranging from intramedullary to extramedullary implants. The purpose of this study was to evaluate the radiological and functional outcomes of 95° dynamic condylar screws in unstable intertrochanteric fractures.

Patients and methods

This is a retrospective review of patients with unstable peritrochanteric fractures (31-A2 and A3 for Association for Osteosynthesis/Association for the Study of Internal Fixation (AO/ASIF) classification) who underwent open reduction and internal fixation with 95° dynamic condylar screw at Recep Tayyip Erdogan Hospital between 2014 and 2018. All the patients had a minimum of one year of follow-up. Clinical outcomes were measured in terms of time to full weight bearing, Harris Hip Score, and time to radiographic union.

Results

This study comprised 84 patients (including 56 males and 28 females with a mean age of 57.2 ± 9.7 years). The average time to full weight bearing was 4.2 months (range: three to six months). The radiological union was seen at a mean of 5.8 months (range: 4-11 months). Functional outcome in terms of Harris Hip Score was graded as excellent, good, fair, and poor in 18, 45, 16, and five patients, respectively. Implant failure was observed in three patients. One patient sustained a peri-prosthetic fracture, which was treated with a longer plate.

Conclusion

Dynamic condylar screw proves to be a reliable implant when used in unstable peritrochanteric fractures and results in satisfactory functional and radiological outcomes.

## Introduction

Increasing life expectancy has led to a considerable increase in the incidence of proximal femur fractures worldwide [1]. These fractures exhibit high morbidity and pose a significant socio-economic burden and therefore are the most important public health issues encountered by orthopedic surgeons [1,2].

Peritrochanteric fractures are classified as stable or unstable. During one-leg standing, stable fractures are less affected by vertical stress, while unstable fractures either exhibit loss of posteromedial buttress or lateral femoral cortex [2,3]. Treatment goals are to obtain stable reduction and fixation of the fracture, allowing the earliest possible mobility [2]. While pre-operatively planning for such fractures, apart from bone quality (osteoporotic vs. normal), several other factors should be taken into consideration such as fracture configuration, comminution, and extension of multiple fracture lines into the femoral neck or subtrochanteric femur [4].

The optimal treatment modality and implant for unstable peritrochanteric fractures are controversial [1,5]. Various implants have been designed to achieve stable fixation goals while simultaneously minimizing complication risk. These implants can be categorized as intramedullary and extramedullary. Intramedullary implants include proximal femur nails and reconstruction nails, whereas extramedullary implants include fixed angle blade plates, 95° dynamic condylar screw (DCS) plates, and proximal femur locking plates [1,6]. For unstable peritrochanteric fractures, multiple clinical trials comparing intramedullary and extramedullary implants reported inconclusive results [2,7]. The purpose of this study was to evaluate the radiological and functional outcomes of 95° DCS in unstable intertrochanteric fractures.

## Materials and methods

This is a retrospective review of patients with unstable peritrochanteric fractures who underwent open reduction and internal fixation with the 95° DCS at Recep Tayyip Erdogan Hospital between 2014 and 2018. Data were retrospectively collected from Indus Hospital’s prospectively maintained electronic medical records database. All patients with unstable peritrochanteric fractures (31-A2 and A3 for Association for Osteosynthesis/Association for the Study of Internal Fixation (AO/ASIF) classification) having a minimum follow-up of one year were included, and those with pathologic fractures, polytrauma, and severe medical co-morbidities to preclude surgery were excluded. Information was collected on patients’ clinic-demographic profiles, e.g., age, gender, mode of injury, and radiographic fracture classification. Clinical outcomes were measured in terms of time to full weight bearing and the Harris Hip Score. Postoperatively, all patients received deep vein thrombosis prophylaxis, and an attempt was made to mobilize them at the earliest. Patients were allowed to bear weight progressively eight weeks post-surgery or after the radiographic appearance of the bony union on at least three cortices. At regular time intervals of six weeks, three months, six months, and one year, patients were called for follow-up. Functional assessment was based on pain, need for analgesics, walking aids, and walking capacity. The clinical union was evidenced by pain-free walking. Radiographic union was based upon bridging callus presence at three of four cortices on orthogonal views.

All data were analyzed using SPSS version 21 (IBM Corp., Armonk, NY). Continuous variables were represented as mean ± SD, and categorical variables were represented as percentages.

## Results

This study comprised 84 patients (including 56 males and 28 females with a mean age of 57.2 ± 9.7 years). The most common injury mechanism was ground-level fall in 63% of patients followed by road traffic accidents (RTA) in 37% of patients. The mean time from injury to surgery was 1.9 ± 1.4 days. The mean blood loss was 395.5 ± 72.6 ml. The mean unit of pack RBCs transfused was 1.32 ± 0.97 units. The mean operative time was 81.7 ± 23.4 minutes. The mean length of hospital stay was 2.8 ± 1.3 days.

All patients had a minimum of one year of follow-up (mean 15.1 ± 2.5 months). The average time to full weight bearing was 4.2 months (range three to six months). The radiological union was seen in 81 out of 84 patients at a mean of 5.8 months (range 4-11 months). In terms of the Harris Hip Score, functional outcome was graded as excellent in 18 patients, good in 45 patients, fair in 16 patients, and poor in five patients. All patients with post-operative poor functional outcomes presented with limited mobility pre-operatively as well.

Superficial surgical site infection was seen in two cases, which resolved uneventfully after local debridement and antibiotics. Acceptable alignment (varus-valgus angulation up to 10° and rotation up to 15°) was achieved in 76 patients (90.5%). The remaining patients presented with malunions that were functionally acceptable (no significant restriction in hip motion and no obvious deformity of proximal femur) and did not require correction.

**Figure 1:**
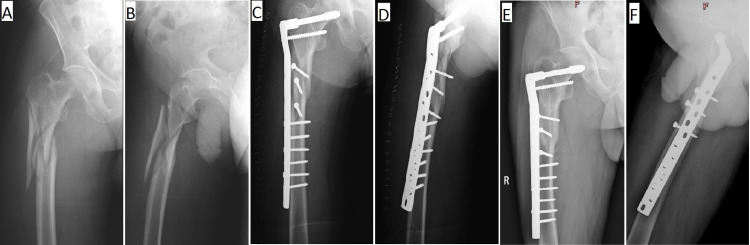
A 58-year-old male with a comminuted peritrochanteric fracture with subtrochanteric extension. A and B: Pre-operative X-rays. C and D: Immediate post-operative X-rays. E and F: One-year follow-up X-rays show a completely healed fracture.

Implant failure was seen in three patients. One patient underwent redo fixation with a longer plate, the second patient received implant removal and fixation with cephalomedullary interlocking nail, whereas the third one was referred to another setup for conversion hemiarthroplasty. One patient sustained a peri-prosthetic fracture, which was treated with a longer plate (Figure 1).

## Discussion

This study’s results show that when used in unstable peritrochanteric fractures, DCS proves to be a reliable implant and results in satisfactory functional and radiological outcomes.

To achieve rigid internal fixation and early mobilization goals [1], the surgeon should familiarize himself with the parameters that contribute to such fractures’ inherent instability and ultimate fixation failure. These factors include fracture comminution leading to loss of posteromedial buttress or burst lateral wall, multiple fracture lines with extension into the adjacent femoral neck or subtrochanteric area or coronal split, and reverse oblique fracture pattern as well as the quality of bone (osteoporotic vs. normal bony architecture) [4,8]. Failure risk can be minimized with proper implant selection, anatomic reduction, and immaculate surgical technique, which preserves fracture biology [4].

For unstable peritrochanteric fractures, multiple implants have been introduced ranging from extramedullary to intramedullary devices over the course of time [2,9], most popular of which is the sliding hip screw [2,9,10]. However, with sliding hip screw use in unstable fractures, unacceptably high failure rates have been reported because the large diameter lag screw does not cross the primary fracture line, and implant telescoping causes medialization of the femur shaft leading to distraction rather than compression at the fracture site [2,9,11].

The 95° DCS was introduced to troubleshoot problems encountered with the sliding hip screw [9,11]. Compared with the blade plate, technical advantages of DCS include the ability to produce stability in the sagittal plane [1]. Conversely, major DCS drawbacks include devascularization of fracture fragments due to extensive dissection leading to delayed union, nonunion, and/or infection [12,13] as well as implant fatigue and failure in the long term [14]. In a study by Sahin et al. [1], one patient out of 37 patients (2.7%) had femoral head perforation. Kulkarni et al. [15] reported an overall implant failure in 11 out of 58 patients. Ten out of 11 young patients united primarily. Out of 47 older patients, 10 patients had implant failure. Restricted weight-bearing status postoperatively was associated with a significantly lower incidence of implant failure (P < 0.05). They recommended that DCS should not be used if weight bearing cannot be minimized in older patients.

To avoid complications associated with DCS, intramedullary devices like proximal femoral nails (PFNs) were devised [9]. Biomechanical advantages of PFN are attributed to its intramedullary nature, which provides a shorter lever arm, better stability against later fracture collapse, and buttress effect against medialization of femur shaft [11,16]. The helical blade of the PFN increases its bone purchase in the femoral neck-head and also prevents rotation or compaction of the proximal fragment by locking with the nail rotationally [17]. Contrarily, major disadvantages of PFN are a difficult closed reduction [18], screw cut-outs, screw migration, peri-implant femoral fractures, malunion, and nonunion [18,19].

There are very few studies comparing intramedullary fixation with angular stable plates for the treatment of unstable fractures [20]. In the previously published literature, investigators stated that fixation with PFN has a shorter operation time, less blood loss, reduced surgical trauma, a shorter hospital stay with earlier weight bearing, lower rates of implant failure, and delayed healing as compared with extramedullary devices [11,16]. However, there is no difference in late functional outcomes when compared with DCS [16]. Cochrane systematic review and meta-analysis conducted by Parker et al. concluded that for stable peritrochanteric fracture, both intramedullary and extramedullary devices achieve good results. For unstable fracture, evidence from available literature is insufficient to favor any implant and more studies are needed for making stronger recommendations. However, they opined that intramedullary implants may yield better outcomes in unstable peritrochanteric fractures [21].

From a developing nation’s perspective, the debate on the most effective treatment modality still revolves around the most cost-effective method of treatment. In a healthcare system where patients themselves are primary payers of medical care services, a cheaper and more widely available DCS implant makes it the most attractive option. Our hospital is a welfare trust setup that offers international standard services free of cost with donations and alms making up for hospital expenses. Therefore, we emphasize that with the use of properly performed techniques, the success rate is high.

The conclusions from this study are limited by the small sample size, retrospective nature, and short follow-up of patients.

## Conclusions

The main objective of the management of elderly patients with peritrochanteric fractures is a successful return to safe mobility. We conclude that with the use of biological (indirect) reduction techniques, DCS proves to be a reliable implant when used in unstable peritrochanteric fractures and results in satisfactory functional and radiological outcomes.
